# Physical Exercise Promotes Recovery of Neurological Function after Ischemic Stroke in Rats

**DOI:** 10.3390/ijms150610974

**Published:** 2014-06-18

**Authors:** Hai-Qing Zheng, Li-Ying Zhang, Jing Luo, Li-Li Li, Menglin Li, Qingjie Zhang, Xi-Quan Hu

**Affiliations:** Department of Rehabilitation Medicine Science, the Third Affiliated Hospital of Sun Yat-sen University, Guangzhou 510630, China; E-Mails: zhangliying_good@126.com (L.-Y.Z.); jill_272@foxmail.com (J.L.); ovelyxt2008@163.com (L.-L.L.); serlin313@hotmail.com (M.L.); zhangqingjie3415@163.com (Q.Z.)

**Keywords:** physical exercise, IGF-1, Akt, apoptosis, neurogenesis, middle cerebral artery occlusion (MCAO)

## Abstract

Although physical exercise is an effective strategy for treatment of ischemic stroke, the underlying protective mechanisms are still not well understood. It has been recently demonstrated that neural progenitor cells play a vital role in the recovery of neurological function (NF) through differentiation into mature neurons. In the current study, we observed that physical exercise significantly reduced the infarct size and improved damaged neural functional recovery after an ischemic stroke. Furthermore, we found that the treatment not only exhibited a significant increase in the number of neural progenitor cells and neurons but also decreased the apoptotic cells in the peri-infarct region, compared to a control in the absence of exercise. Importantly, the insulin-like growth factor-1 (IGF-1)/Akt signaling pathway was dramatically activated in the peri-infarct region of rats after physical exercise training. Therefore, our findings suggest that physical exercise directly influences the NF recovery process by increasing neural progenitor cell count via activation of the IGF-1/Akt signaling pathway.

## 1. Introduction

Ischemic stroke, one of the most serious neurological disorders, causes permanent impairment in patients, affecting their motor and cognitive-communication abilities and can lead to learning and memory deficits [1,2]. Despite clinical and technological advances over recent decades in the understanding and treatment of ischemic stroke, these dysfunctions significantly reduce the quality of daily life for patients [2,3,4]. However, recent rodent studies have indicated that physical exercise acts as an effective rehabilitation program for treatment of ischemic stroke [5,6] by promoting the recovery of the sensory-motor function and preventing deterioration in cognitive ability [7]. Similarly, Lee *et al*., have reported that moderate activity can protect against brain injury after a stroke [8]. While the positive effects of early exercise on functional outcome after stroke are widely recognized, their underlying mechanisms are poorly understood.

Recently, multiple growth factors, such as insulin-like growth factor-1 (IGF-1), have been reported to provide beneficial effects on functional recovery in cerebral ischemia [9,10]. A study by De Smedt and co-workers found that the serum levels of IGF-I in patients with ischemic stroke showed a significant correlation with functional recovery [11]. Furthermore, Russo *et al*., have reported that IGF-1 plays a pleiotropic role within the central nervous system, preventing specific processes from occurring, including the stimulation of the proliferation; differentiation of neural progenitor cells; protection of neuronal and glial cell from apoptosis; modulation of inflammation; and inhibition of excitotoxicity [12]. It has been reported that physical exercise improved the function of ischemic brain by increasing circulating levels of IGF-1 [9,10], but the effect of physical exercise on local production of IGF-I has not been comprehensively studied. The present study focuses on the promotion of neurogenesis and neural progenitor cell count, attenuation of apoptosis and the impact of exercise treatment on the activation of IGF-1/Akt signaling in order to further investigate the physical exercise related protective mechanism and its effect on neural-injury induced by ischemic stroke.

## 2. Results

### 2.1. Physical Exercise Improves Recovery of Neurological Function (NF)

**Figure 1:**
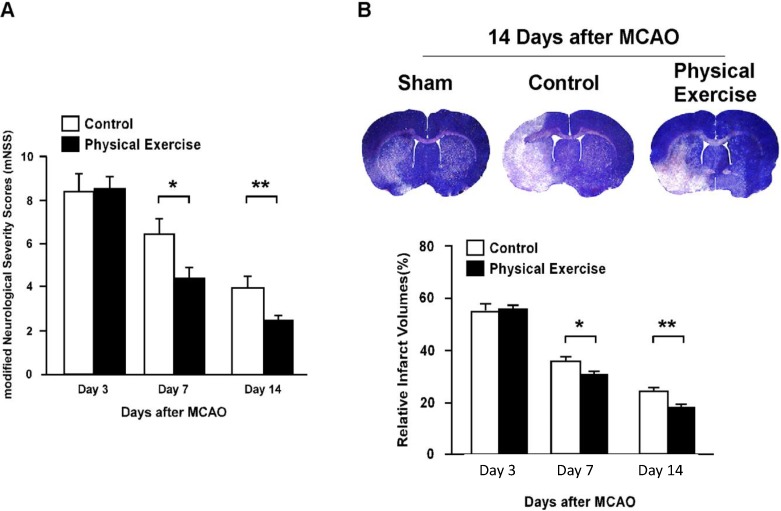
Neurological function (NF) scores, Nissl staining and the infarct areas of the control and physical exercise groups. (**A**) Neural function was evaluated after middle cerebral artery occlusion (MCAO) using the modified Neurological Severity Scores (mNSS). The scores in the physical exercise group were significantly lower in comparison to the control; (**B**) (**Upper panel**) Nissl staining of brain tissues at day 14 after transient MCAO and (**Lower panel**) the quantification of the relative infarct volume in physical exercise group *versus* the control group (* *p* < 0.05; ** *p* < 0.01).

In order to explore the mechanism of physical exercise on neuron-protection, the middle cerebral artery occlusion (MCAO) assay was established. Neurological function (NF) after an ischemic stroke was evaluated using the modified Neurological Severity Scores (mNSS) scale at days 3, 7 and 14, in rats from the control and exercise groups. The mNSS in the control group were determined as 8.4 ± 0.8 on day 3, 6.5 ± 0.7 on day 7 and 4.0 ± 0.5 on day 14, respectively (Figure 1). Interestingly, the rats subjected to exercise displayed an average mNSS of 8.5 ± 0.6 on day 3, which was comparable to the control group. However, a significant decrease was observed on days 7 and 14, yielding 4.4 ± 0.5 and 2.5 ± 0.2 (*p* < 0.05; *p* < 0.01), respectively (Figure 1A). Meanwhile, no marked alertness of mental wellbeing were observed in tested rats, which included general activity levels, coat condition and posture, mean arterial pressure (MAP), rectal temperature (T), heart rate (HR), arterial blood gas values (ABGV), venous blood glucose levels (Glu) and body weight (W) (Table S1). Therefore, our results suggest that physical exercise improved the recovery of NF after an ischemic stroke.

### 2.2. Physical Exercise Reduces the Volume of the Infarct Area after an Ischemic Stroke

To further probe the effects of physical activity, the relative cerebral infarct volume after MCAO was examined. As shown in Figure 1B, the infarct volume on day 3 was 54.91% ± 0.7% for the exercised group, whereas the control displayed 55.04% ± 0.82% (*p* = 0.064). Compared to the control, exercise significantly reduced the infarct volume on day 7 (35.58% ± 1.67% *vs*. 33.48% ± 1.39%), in the non-exercise and exercise groups, respectively, *p* < 0.05) and 14 (23.83% ± 0.94% *vs*. 17.355% ± 0.57%, *p* < 0.01). The results indicated that physical exercise was able to reduce the infarct volume significantly.

### 2.3. Physical Exercise Increases Newborn Neurons and Neural Progenitor Cells

**Figure 2:**
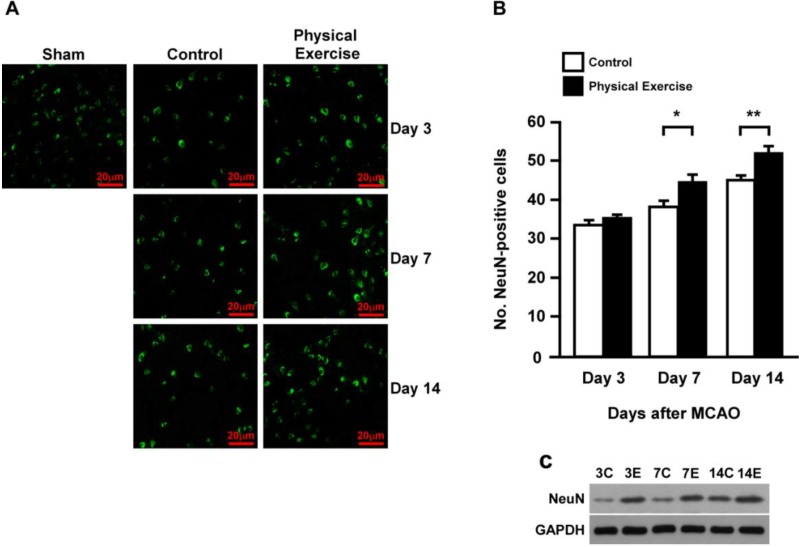
Physical exercise increases the new neuron count in the peri-infarct region after ischemic stroke. Expression of NeuN-positive cells in the peri-infarct region for both the physical exercise (E) and control groups. NeuN immuno-staining (**A**,**B**); and Western Blotting (**C**) analyses in the physical exercise group was significantly higher than that in the control group at days 7 and 14 after MCAO, while no disparity was observed between the groups at day 3 (* *p* < 0.05; ** *p* < 0.01).

**Figure 3:**
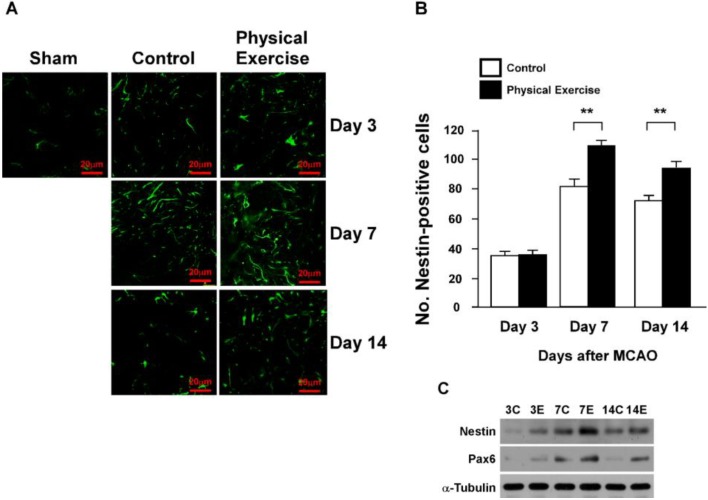
Physical exercise increases the neural progenitor cell count in the peri-infarct region after an ischemic stroke. Expression of Nestin-positive cells in the peri-infarct region. Immuno-staining (**A**,**B**) and Western Blotting (**C**) analyses were performed. The expression levels of nestin and Pax6 in the physical exercised group were significantly higher than that in the control group at days 7 and 14 after MCAO, while no disparity were observed between the groups at day 3 (** *p* < 0.01). The results of westerns for Nestin and Pax6 are representative of three independent experiments.

As expected, we found that the mature neuron count in the peri-infarct region of the exercise group was considerably higher than that in the control group, as indicated by NeuN immuno-staining and western blotting analyses at days 7 and 14 (*p* < 0.05; *p* < 0.01) (Figure 2A–C). Previous studies have demonstrated that neural progenitor cells play a vital role in NF recovery by differentiating into mature neurons [13]. Therefore, we examined the expression of Nestin, which is a neural progenitor cell-marker for such precursors. As shown in Figure 3A, we observed an apparent increase in the Nestin-positive cell count in both the exercise and control groups. In comparison, the sham-operated group (after MCAO) exhibited no change in cell count at day 3. However, the cells with high Nestin-expression in the exercise rat group was appreciably higher than in the control group at days 7 and 14 (*p* < 0.01; *p* < 0.01), which further confirmed by the expression of Pax6, another identified neural progenitor cells (NPCs) marker (Figure 3A–C). The combination of these results suggested that exercise may increase the number of new neurons and neural progenitor cells in the peri-infarct region after an ischemic stroke.

### 2.4. Physical Exercise Decreases Apoptosis in the Peri-Infarct Region

**Figure 4:**
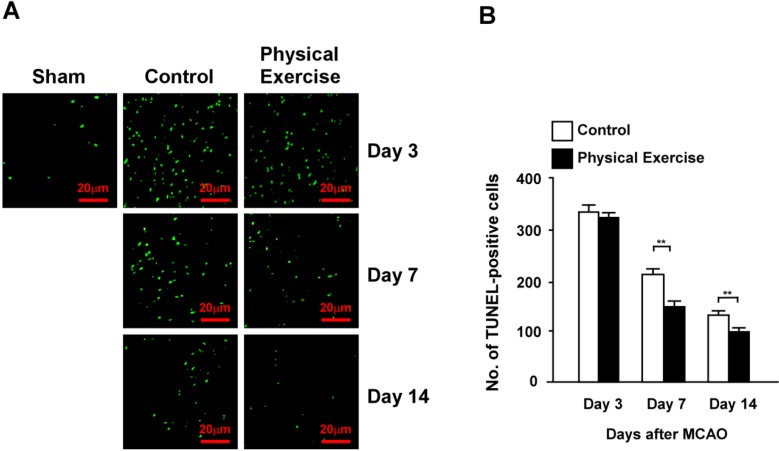
Physical exercise decreases apoptosis in the peri-infarct region after an ischemic stroke. (**A**,**B**) Expression of transferase-mediated dUTP *in situ* nick-end labeling (TUNEL)-positive cells in the peri-infarct region. TUNEL-positive cell count in the physical exercise group was significantly lower than that observed in the control group at days 7 and 14 after MCAO, while no disparity was observed between the groups at day 3 (** *p* < 0.01).

No obvious differences were observed in the transferase-mediated dUTP *in situ* nick-end labeling (TUNEL)-positive signals between the exercise and control groups at day 3 (*p* > 0.05). In contrast, the TUNEL-positive cell count in the exercise group was significantly less than in the control group for both days 7 and 14 (*p* < 0.01, *p* < 0.01, respectively) (Figure 4A), implying that physical exercise decreased apoptosis in the peri-infarct region following ischemic stroke.

### 2.5. Physical Exercise Activates Insulin-Like Growth Factor-1 (IGF-1)/Akt Signaling in the Peri-Infarct Region

**Figure 5:**
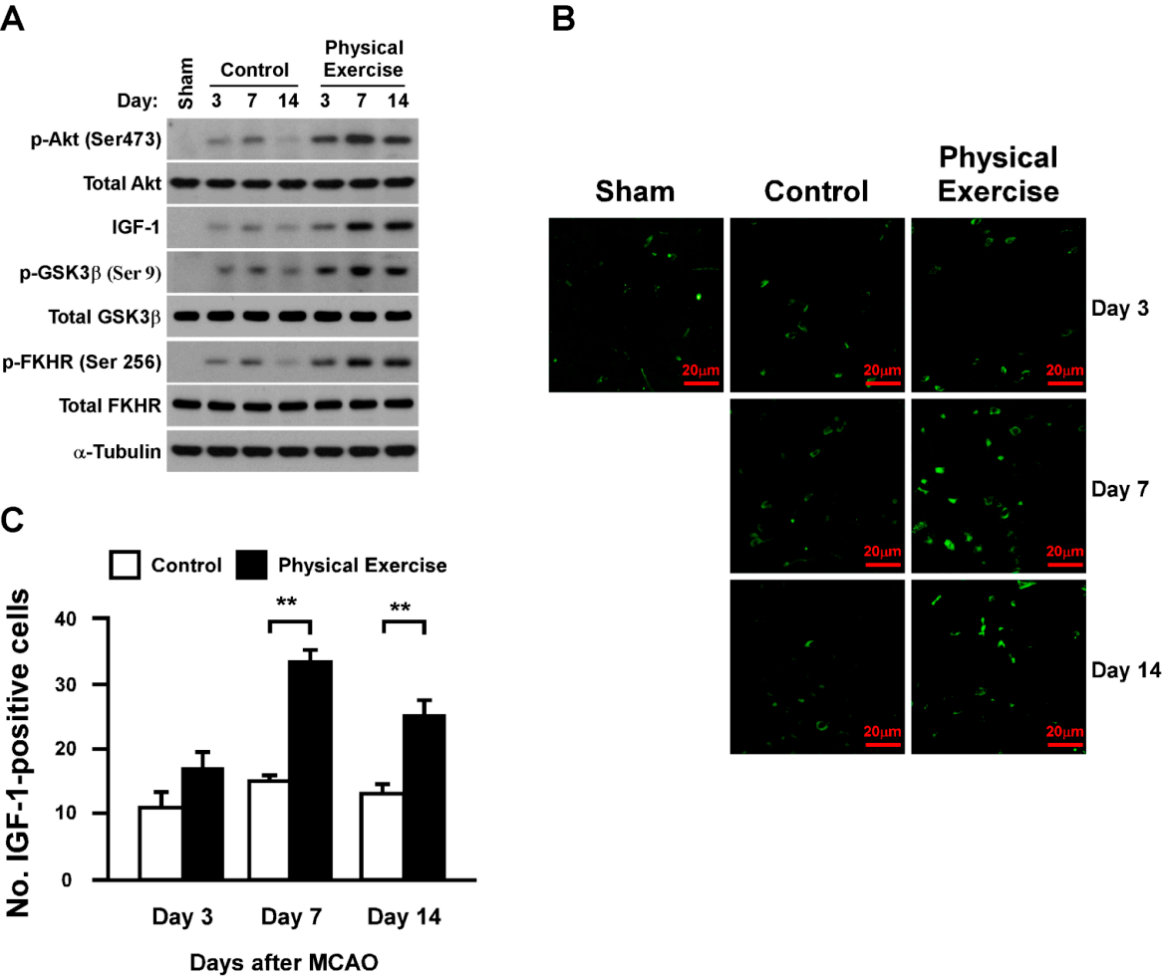
Physical exercise activates IGF-1/Akt signaling in the peri-infarct region after an ischemic stroke. (**A**) The expression of p-Akt/total-Akt, p-GSK-3β/GSK-3β, p-FKHR/FKHR (fork-head transcription factor) and IGF-1 in the peri-infarct region analyzed by western blotting. Protein expression levels were normalized with α-Tubulin; (**B**,**C**) Expression and quantification of IGF-1-positive cells in the peri-infarct region. IGF-1-positive cell count in the physical exercise group was significantly higher than that observed in the control group at days 7 and 14 after MCAO, while no disparity was observed between the groups at day 3 (** *p* < 0.01).

It has been demonstrated that Akt signaling plays a key role in cell survival and anti-apoptosis [14,15], which encouraged us to investigate the capacity of physical exercise to activate the Akt pathway. Indeed, we found that the expression levels of p-Akt (Ser 473) increased dramatically in the exercise group on days 7 and 14 compared with those in the control group (Figure 5A). Consistently, the phosphorylated-GSK (Glycogen synthase kinase)-3β and -FKHR (fork-head transcription factor), the downstream targets of Akt, were also dramatically increased in the exercise group (Figure 5A). Notably, we also discovered that physical exercise drastically increased the expression of IGF-1, a key upstream growth factor of Akt signaling (Figure 5A). Consistently, immuno-staining assays confirmed that the IGF-1-positive cell count in the exercise group was substantially higher than that in the control group on days 7 and 14 (*p* < 0.01, *p* < 0.01, respectively) (Figure 5B,C). The outcomes of this study reveal that physical exercise markedly induces IGF-1/Akt signaling activation after an ischemic stroke.

### 2.6. Discussion

#### 2.6.1. Physical Exercise Promotes Nestin- and NeuN-Positive Cells, and Attenuates Apoptosis in the Peri-Infarct Region

In the current study, we observed that physical exercise could promote NF recovery and reduce the infarct volume following an ischemic stroke. Nestin and NeuN immuno-staining revealed that the neural progenitor cell and neuron counts increased significantly in both the control and exercise groups, whereas the apoptotic cell count decreased only in the exercise group. Importantly, immunochemistry and Western Blotting showed that the IGF-1/Akt signaling pathway was also dramatically activated in the exercise group. Therefore, our findings indicate that physical exercise plays a critical role in NF recovery, presumably through activation of the IGF-1/Akt signaling pathway, enhancement of the neural progenitor cell count and attenuation of apoptosis.

It has also been reported that NPCs, generated from the subventricular zone (SVZ), could migrate towards the peri-infarct region, and differentiate into neural cells possibly improving neurological function after ischemic stroke [16]. Furthermore, Thored *et al*., suggested that stroke-induced neurogenesis is extensive and long-lasting, with continuous production of mature striatal neurons for several months after the insult [17]. However, Yamashita *et al*. demonstrated that the number of newborn neurons is too low for recovery of neurological functions, and it is necessary to add appropriate interventions to enhance the proliferation, survival, and/or neuronal maturation of newborn cells [13]. Physical activity, a robust stimulus of adult neurogenesis at the behavioral level, promotes the proliferation of neural stem cells (NSCs) and the survival and maturation of newborn neurons within the dentate gyrus of the hippocampus [18]. We herein observed that NF recovery has been improved and infarct volume decreased in the physical exercise group. In our current study, we found that the numbers of NPCs and neurons dramatically increased after stroke. Moreover, we found that physical exercise promoted the expression of Nestin- and NeuN-positive cells and attenuated apoptosis in the peri-infarct region. Therefore, these results suggest that the exercise-induced promotion and survival of neurons contributes to the improvement of rat behavior and brain structure [16].

#### 2.6.2. Physical Exercise Can Promote the Expression of IGF-1 in the Peri-Infarct Region

Previously, multiple growth factors, including insulin-like growth factor-1 (IGF-1), have been reported to provide beneficial effects on functional recovery in cerebrasal ischemia [9,10]. Several studies have demonstrated that activation of Akt signaling may promote cell survival through phosphorylation of multiple substrates, such as glycogen synthase kinase 3β (GSK-3β) and fork-head transcription factor (FKHR) [14,15]. Accordingly, Chung *et al*., have reported that a specific ghrelin hormone increases the cellular proliferation of adult rat hippocampal neural stem cells (NSCs) by influencing the activation of the pathway [19]. Moreover, Rahmani and colleagues found that continuous fluoxetine treatment elevates the survival rate of differentiated neural cells and promote neurogenesis through induction of Akt phosphorylation [20]. A more recent report relating to ischemia has also highlighted this pathway resulting in the promotion of neuron survival [21].

In order to investigate the effected factors of exercise-induced improvement after cerebral ischemia, the expression of IGF-1/AKt pathway were further detected in the present study. We found that exercise increased the expression of phosphorylated-Akt (Ser 473), indicating that the Akt pathway is activated in the peri-infarct region after an ischemic stroke. Hence, further investigation of downstream targets of Akt might shed light on the observed outcomes of exercising, which could be achieved using Akt kinase that was activated through phosphorylation by membrane-bound receptor tyrosine kinase (RTK) via stimulation of growth factors, including IGF-1 [14,22,23]. Previous studies have reported that exercise prevents the occurrence of brain damage through an increased uptake of circulating IGF-1. Importantly, the administration of a blocking antibody (anti-IGF-1) to the exercising animals inhibits this uptake and abrogates the protective effects of exercise in all types of lesions [9,10].

As mentioned above, IGF-1 plays a key role in central nervous systems by stimulating neurogenesis, differentiation of neural progenitor cells, and protection of neuronal and glial cells from apoptosis [12]. Moreover, Arsenijevic *et al*., have demonstrated that IGF-1 is necessary for stem cell proliferation and required for the induction of epidermal (EGF) and fibroblast (FGF-2) growth factors in striatal stem cell cultures [24]. Carlos and co-workers have reported that endogenous IGF-1 is required for differentiation of olfactory bulb stem cells/precursor cell-derived neurons and glia both *in vitro* and *in vivo*, which suggests that exogenous IGF-1 can act in an autocrine/paracrine manner, thus functioning as a differentiation and/or survival factor [25]. IGF-I is expressed in neurons and microglial cells [12], and IGF1 receptor (IGF1R) immunoreactivity was predominantly expressed by microglia [26]. Such studies indicate that IGF-1 participates in the promotion of *in vivo* neurogenesis. Consistently, we also found that the local production of IGF-I was induced by physical exercise and the IGF-1-positive cells were evidently increased in the ischemic cortex but were rarely detected in the contra-lateral hemisphere. Taken together, our results suggest that the IGF-1/Akt pathway contributes to the promotion and survival of neural cells in cerebral ischemia.

## 3. Experimental Section

### 3.1. Animals and Treatments

All experimental procedures were approved by the Institutional Animal Ethical Committee of Sun Yat-sen University and were conducted according to the Guide for the Care and Use of Laboratory Animals of the National Institute of Health (Publication No. 80–23, revised 1996). A total of 40 male Sprague-Dawley rats weighing 250–280 g were used for this experiment. Rats were housed in the same animal care facility during a 12 h light/dark cycle throughout the protocol. Subsequently, the rats were subjected to transient focal cerebral ischemia induced by left-transient middle cerebral artery occlusion (MCAO) as described previously [27,28,29]. Briefly, rats were anesthetized with an intra-peritoneal injection of 3.5% chloral hydrate (350 mg/kg) and were placed in the supine position. Body temperature of the rats was maintained at 37 ± 0.5 °C, on a heating pad. The left common carotid artery (CCA), internal carotid artery (ICA), and external carotid artery (ECA) were surgically exposed. The CCA was ligated distally and the ECA was ligated proximally to the bifurcation of the ICA and the ECA. A 3–0 silk suture was tied loosely around the ICA, and a micro vascular clip was placed distally across the ICA. A filament (4–0 nylon suture with a rounded-tip) was inserted into the ICA through the CCA and gently advanced from the CCA bifurcation to block the middle cerebral artery (MCA) at its origin. The suture around the ICA was tightened, and the micro vascular clip was removed. Mean arterial blood pressure, heart rate and arterial blood gases were analyzed during the process of surgery. The suture was pulled back until the tip reached around the ICA to restore blood flow (re-perfusion) after 90 min of MCAO. Following wound closure, the animals were allowed to recover from anesthesia and subsequently housed in cages.

The NF was evaluated using the Bederson’s neurological function test, after the 6 h MCAO. The Bederson’s scores are as follow: no deficits score, 0; unable to extend the contra-lateral forelimb score, 1; flexion of contra-lateral forelimb score, 2; mild circling to the contra-lateral side score, 3; severe circling and allying to the contra-lateral side score, 4. The rats with scores 1–3 were selected and randomly divided into three groups: the physical exercise group (*n* = 15), which was subjected to a running exercise each day for 3 days after transient MCAO. The control group (*n* = 15), and sham-operated group (*n* = 5, where the filament was not inserted into the artery) were fed in standard cages with no additional exercise and served as controls. To normalize for handling stress, sedentary animals in the control and sham-operated groups were placed on non-moving wheels for a time duration that was equivalent to the exercise treatment. Exercised rats were further randomized into one of three groups with different exercise durations of either 3, 7 or 14 days, after transient MCAO. Correspondingly, sedentary rats were also randomly divided into three groups under 3, 7 and 14 days following transient MCAO.

### 3.2. Exercise Training and Function Testing

The exercise regime was chosen based on the protocol according previous report [30]. All animals subjected to the running wheel exercise were placed into a programmable, motorized wheel apparatus (21 cm diameter, 40 cm length, made in South China University of Technology, Guangzhou, China), permitting the quantification of exercise intensity. Rats in the physical exercise group were placed into the wheel to run for 3 days post-ischemia. Initially, the running speed was set at 5 rev/min (approximately 3 m/min), for 20 min, twice a day (morning and afternoon), then gradually increased to 10 rev/min (approximately 6 m/min) on day 7, followed by 15 rev/min (approximately 10 m/min) on day 10. The control and sham-operated groups were housed in a standard cage (*n* = 5) with free access to food and water no specific exercise. The distance traveled at each time-point was showed in Table S2. The body weight of each rat was monitored every 3 days. During the experiments, rats did not run when first put in the running wheel, but ran after repeated placements in running wheel; none of the animals fell off the wheel in our study. No animals died before the planned study endpoint.

All rats in this study were given 1 week pre-conditioning exercise before MCAO and subsequently the NF of each rat was assessed on a scale of 0–18 (normal score, 0; maximal deficit score, 18) [31,32]. Neurological severity score is a combination of test for motor, sensory, reflex, and balance [33].

### 3.3. Tissue Preparation for Histochemistry

Rats were sacrificed after the completion of the motor functional evaluation on days 3, 7 and 14 after transient MCAO (*n* = 5 per group at each time point) with an overdose of 10% chloral hydrate and perfused transcardially with 0.9% saline at 4 °C, followed by 4% paraformaldehyde in phosphate buffer (0.1 mol/L, pH 7.4) [11]. The brains were removed, fixed in the above fixative for 8 h at 4 °C, and then immersed sequentially in 20% and 30% sucrose until sinking occurred. Coronal sections (10 μm thick) were cut on a cryostat (CM1900; Leica, Heidelberger, Germany) from bregma +4.0 to −6.0 mm and used for Nissl, immunoflourescence and TUNEL staining.

### 3.4. Nissl Staining

Serial sections from bregma +4.0 to −6.0 mm were selected for Nissl staining to measure the infarct volume in the ipsilateral hemisphere. Nissl staining was performed with 0.1% cresyl violet (Sigma, Saint Louis, MO, USA) according to a previously reported procedure [3]. For quantification of infarct volume, five successive coronal sections at 2.0 mm intervals from bregma (+4.0 to −6.0 mm) were selected. The infarct areas are referred to as zones of irreversible ischemic damage, which have been exposed to extremely severe reduction in cerebral blood flow and exhibit severe, consistent, ischemic damage. The area immediately outside of this zone is referred to as the “peri-infarct”, in which most neurons do not display the histological signs of irreversible damage [34]. The volumes of the ipsilateral and the contra-lateral hemisphere were counted as described previously [3,35,36] and the relative infarct volume was described as a percentage of the contra-lateral hemisphere. All the mNSS, infarct size calculation and immunostaining quantitation were performed blinded, which were reviewed and analyzed separately by two independent researchers.

### 3.5. Immunofluorescence Staining

Immunofluorescence staining of Nestin, NeuN and IGF-1 were performed using the following methods according previous report [37]. In brief, serial sections from bregma 0.20 to −2.20 mm were selected by a research assistant blind to the study protocol. The sections were pre-treated for 5 min with hot (85 °C) citrate buffer (0.01 mol/L, pH 6.0) for antigen retrieval, followed by 5% normal goat serum for 1 h at room temperature. Next, the sections were incubated with mouse anti-Nestin (1:200; Millipore, Billerica, MA, USA), mouse anti-NeuN (1:200; Millipore), or mouse anti-IGF-1 (insulin-like growth factor, 1:100; Millipore) overnight at 4 °C. After rinsing in phosphate-buffered saline (PBS) 3 times in 5 min cycles, the sections were incubated with peroxidase-marked mouse secondary antibody (anti-mouse IgG, 1:1000, Cell Signaling, Danvers, MA, USA) for 1 h at room temperature. Fluorescence signals were detected with an optical microscope (BX51; Olympus, Tokyo, Japan) under the same exposure. The negative control sections were incubated with PBS instead of primary antibodies and showed no positive signals in fluorescence.

All histological images captured in the same exposure were analyzed with the Image-Pro Plus analysis software (Media Cybernetics, Silver Spring, MD, USA). Eight consecutive sections were analyzed at 240 μm intervals from bregma (0.20 to −2.20 mm). The cell count was collected using four non-overlapping fields (425 μm × 320 μm) under an optical magnification of 400× and was presented as the average cell number per field on each section and ten representative staining fields of each sample were analyzed.

### 3.6. Deoxynucleotidyl Transferase-Mediated dUTP in Situ Nick-End Labeling (TUNEL) Assay

TUNEL assay was performed in the peri-infarct region, using an *in situ* cell death detection kit (Fluorescein; Roche Corp., Basel, Switzerland), according to the manufacturers’ protocol. TUNEL signals were observed with an optical microscope (BX51; Olympus). Visible TUNEL-positive cells were counted in each group.

### 3.7. Western Blotting Analysis

Rats were heavily anesthetized with 3.5% chloral hydrate and the rat brains were quickly extracted after sacrifice and sliced into 5 mm × 2 mm thick sections (rostro-caudal direction, starting at 1 mm from the rhinal fissure) and immersed for 5–10 min in a saturated solution of Triphenyl Tetrazolium Chloride in 1× phosphate-buffered saline to stain dead cells in white, which indicated the infarct region. Furthermore, a 2 mm strip adjacent to infarct peripheral edge, as “peri-infarct region”, were collected for western blotting analysis [38,39]. Rat brains were dissected and homogenized in lysis buffer. The lysate was then centrifuged at 12,000× *g* for 5 min. The supernatant protein concentration was measured with the Bradford Protein Assay, by heating the supernatant at 95 °C for 5 min. 30 mg of the resulting sample was loaded onto a 10% polyacrylamide gel and separated at a voltage of 120 V. Proteins from the gel were then transferred onto a polyvinylidene difluoride (PVDF) membrane. The membranes were incubated overnight at 4 °C with anti-IGF-1, anti-Pax6, anti-Nestin, and anti-NeuN (Millipore), and anti-p-Akt, anti-GSK-3β and anti-FKHR antibodies (Cell Signaling, Danvers, MA, USA), rinsed with Tris Buffered Saline Tween (TBST) and then incubated with anti-mouse IgG (1:1000 dilution; Cell Signaling Technology) for 1 h at room temperature. To confirm equivalent loading of samples, the same membranes were incubated with α-Tublin antibody. The immune complexes were detected by emitter-coupled logic (ECL, Cell Signaling Technology) and exposed to X-ray film. The results of Westerns Blotting were representative of three independent experiments.

### 3.8. Image Analysis and Quantification

All histological images were analyzed with the Image-Pro Plus analysis software (Media Cybernetics, Silver Spring, MD, USA). The regions of interest (referred to as “zones”) with dimension 700 μm × 700 μm (width and length), were selected from the peri-infarct region, immediately outside of the infarct zone. For the Nestin/NeuN/IGF-1/TUNEL-immunopositive cell counts, eight consecutive sections were analyzed at 240 μm intervals from bregma (0.20 to −2.20 mm). The cell count was collected by Image-Pro Plus image analysis software using four non-overlapping fields (425 μm × 320 μm) under an optical magnification of 400× and was presented as the average cell number per field on each section [36,37]. The final cell count per rat was the average cell count of all sections examined. All the mNSS, infarct size calculation and immunostaining quantitation were performed blinded, which were reviewed and analyzed separately by two independent researchers.

### 3.9. Statistical Analysis

The Pearson bivariate correlation procedure was used to run the correlation analysis. Statistical analysis was performed using the SPSS 16.0 statistical software package (SPSS Inc., Chicago, IL, USA). Two independent samples *t*-test were employed for 2-group comparisons for the number of Nestin/NeuN/IGF-1/TUNEL-positive cell variables. Repeated measures ANOVA were used to evaluate the modified Neurological Severity Score (mNSS) variables. A non-parametric test was then applied to determine the mNSS and infarct volumes. Numerical data were presented as a mean ± SD. * *p* < 0.05, ** *p* < 0.01, *** *p* < 0.001.

## 4. Conclusions

In summary, our results suggest that physical exercise plays a key role in NF recovery, by promotion of neurogenesis via activation of the IGF-1/Akt signaling pathway. Further studies are needed to investigate the detailed mechanism of exercise induced-IGF-1 expression. Although physical exercise-promoted NF recovery has been extensively studied, the underlying protective mechanisms are still not well understood. Therefore, identification of potential factors and clarification of their contributions to the promotion of neurogenesis may provide further insight into the biological basis of NF recovery.

## Supplementary Files

Supplementary File 1 Supplementary Information (PDF, 555 KB)
