# Supplementary material for: Physical Exercise Promotes Recovery of Neurological Function after Ischemic Stroke in Rats
Source: Int J Mol Sci. 2014 Jun 18;15(6):10974–88. doi: 10.3390/ijms150610974 (PMC4100192; doi:10.3390/ijms150610974)
Supplement: Supplementary File 1 — Supplementary Information (PDF, 555 KB) [file ijms-15-10974-s001.pdf]

Supplementary Information

Table S1. Physiological alteration in tested rats.

Physiological Indicators	Sham	Control	Physical Exercise
MAP (mmHg)	120 ± 12.3	122 ± 11.6	125 ± 12.0
HR (f/min)	419 ± 19	421 ± 23	427 ± 21
W (g)	268 ± 11	265 ± 13	267 ± 14
T (°)	37 ± 0.5	37 ± 0.5	37 ± 0.5
Glu (c/mmol·L^{-1})	8.41 ± 1.03	8.24 ± 1.21	8.38 ± 1.43

Table S2. Training distance.

Training Time	Sham	Control	Physical Exercise
Day 3	quiescence	quiescence	run 120 m
Day 7	quiescence	quiescence	run 720 m
Day 14	quiescence	quiescence	run 2400 m

©2014 by the authors; licensee MDPI, Basel, Switzerland. This article is an open access article distributed under the terms and conditions of the Creative Commons Attribution license (http://creativecommons.org/licenses/by/3.0/).
